# New Elements of Operative Surgery

**Published:** 1845-03

**Authors:** 


*New Elements of Operative Surgery.* By ALF. A.L. M. VELPEAU, Professor of Surgical Clinique of the Faculty of Medicine of Paris, Surgeon of the Hospital of La Charité, Member of the Royal Academy of Medicine, of the Institute &c. *Carefully revised, entirely remodelled, and augmented with a treatise on minor Surgery. Illustrated by over* 300 *engravings, incorporated with the text; accompanied with an Atlas in quarto of twenty-two plates, representing the principal operative processes, surgical instruments, &c. First American, from the last Paris edition.* Translated by P. S. TOWNSEND, M. D., late Physician to the Seamen's Retreat, Staten Island, New York. *Augmented with the addition of several hundren pages of entirely new matter, comprising all the latest improvements and discoveries in Surgery in America and Europe, up to the present time. Under the supervision of, and with notes and observations by* VALENTINE MOTT, M. D., Professor of the Operations of Surgery with Surgical and Pathological Anatomy, in the University of New York; Foreign Associate of the Académie Royale de Medicine of Paris, of that of Berlin, Brussels, Athens, &c. In three volumes. Vol. 1. New York: Henry G. Langley, 1845.

What a title page! However, *De gustibus*, &c. Authors, translators, editors, and publishers, surely may exercise the privilege of taste, equally with the rest of the world.

Professor Velpeau calls his work "a didactic treatise on operative surgery." It is in fact an abridged history of the operations of surgery, in which every thing, good, bad, and indifferent, is piled up, it must be confessed, in some confusion. However valuable such a treasure is for consultation and reference, we could have wished, for the sake of the student at least, that more order had prevailed, or, at any rate, that the information in reference to operations had been presented to the reader unencumbered by the historical details; which are admitted to be imperfect, and, we may add, too frequently are erroneous. The former is the really important part, while the latter constitutes no inconsiderable share of the present volume.

This, which may be called the *great* work of Velpeau, consists, in the original, of four volumes, but in the American edition, is to be comprised in three, the first of which is now published. The arrangement of the materials of this first part is certainly very *peculiar*, and will afford some idea of the *method* of the work. First we have an *appendix* of twenty-one pages, *preceding* the subject matter of the work; in the middle we have an "*appendix* to tenotomy;" and at the conclusion, another *appendix*, occupying 175 pages. Then we have *Dr. Townsend's* preface, *Dr. Mott's* preface, and the *Author's* preface to the *first*, and his preface to the *second* edition. These, with sundry other matters and the text, make a volume of 851 pages, large octavo.

The most serious fault we have to find, however, with the arrangement of the matter is, the neglect to distribute the items contained in the appendixes under their proper heads throughout the volume,—the concluding appendix, particularly, which contains much valuable information in reference to operations performed in this country. Instead of the whole being collected together at the end of the volume, the information should have been put in its proper connection with the text. By this means all the information on particular subjects would have been presented consecutively, and much trouble and confusion to the reader avoided.

It is true, the translator apologises in a measure for this irregularity, in the following note: "Many things of interest, and some of much importance in surgery, having occurred while this first volume of this American edition has been in the press, we take the occasion, in a concluding appendix, to subjoin whatever has reached us of particular value of this kind to the student or surgeon, and which directly appertains to the matters treated of in this volume; more especially to those new, useful, and attractive departments of modern surgery, which constitute, happily, the leading feature of this portion of M. Velpeau's great work,—we mean *anaplasty* and *tenotomy;* in the contributions to which our own American Surgeons, as will be seen by the liberal citations we have made from Professor Pancoast's, Professor Mütter's, and other publications recently issued from the press, furnish a very considerable and creditable amount," (page 675.) Still we cannot help thinking that much of the new matter thus referred to, might readily have been incorporated as we have suggested.

The translator informs us that he has adhered "as closely and rigidly as possible to the original text of the author, *verbum verbo,* at the risk of appearing, at times, to be speaking in an idiom somewhat Gallican." It is certainly praise-worthy in a translator to adhere strictly to the matter, and as far as possible to the style of his author, but it can hardly be pretended that this is best accomplished by rendering the text so strictly literal as to violate the idiom of the language employed, and certainly when terms are retained *untranslated,* the reader, as well as the author, has a just right to complain. Of this, we have observed frequent instances in the present publication. But when the translation is attempted, we can see no reason why the text should be burthened with words and phrases of the original language, thrown in parenthetically, as in the following example, which, we are sorry to say, is common throughout the volume: "We have cauteries of a reed shape *(en roseau)* a sort of cylindrical rods that may be applied to the deepest passages; the olive shaped, *(en olive,)* which serve for burning the interior of certain cavities and cysts, and the bottom of small excavations. The *conical* cautery is more particularly designed to penetrate through a certain quantity of tissues. The *hastile* or *cutelaire* cautery is a species of shield, *(rondache,)* or sapeur's hatchet, designed for making burnt lines, *(des raies de feu)* upon the integuments. When we wish to cauterise flatwise and upon a large surface, we employ the nummulary *(nummulaire)* cautery. That which Percy has described under the name of the *annular* cautery is not used; but we sometimes employ the *bird-beaked, (bec d'oiseau,)* and the "*haricot* cautery." (Page 303.)

The constant introduction of French terms and phrases, which occur throughout the work, would not indicate that confidence n his understanding of their meaning implied in the preface of the translator, as well as in that by Dr. Mott; nor can we divine why proper names of English and American origin should be given in a foreign dress,—such, for instance, as "M. A. Cooper," "M. R. Barton," "M. Earle," "M. Rogers," &c., instead of Sir Astley Cooper, Dr. Barton, Mr. Earle, Dr. Rogers, &c. However, we have no wish to be hypercritical, and with such exceptions as we have mentioned, and which we cannot but regard as great blemishes, the translation is a very fair one—represents the author's meaning well, and is commendably free from grammatical errors.

In his capacity of editor as well as translator, Dr. Townsend has added to the original work considerably, especially in what relates to the operations by American surgeons. We are pleased to observe in this department of the work a very proper disposition to award credit to those to whom it is due, and although we discover many errors, they do not appear to have originated with Dr. T. From the relation existing between the principal translator and Doctor Mott, and the connection of the latter with the present publication, as may naturally be supposed, the opinions and doings of the New York Professor occupy a prominent and considerable part in the account of American surgery. Considering also that it was the object and ambition of Dr. Mott, in taking part in the labor of introducing Velpeau to American readers, as avowed in his letter to the author, to "enter his name as a *compagnon de voyage*" with him "upon the great high road of the science," this was to be expected.

We have said nothing of the doctrines or of the practice promulgated in this volume. Our strictures relate altogether to the *manner* in which it is brought forth. When the whole shall be published, we shall endeavour to find space for a comprehensive notice of the more interesting points embraced in both the original and added matter. For the present we take leave of the work, with the expression of our thanks to the translators for the service they have rendered to the profession in this country, by their laborious undertaking; and to the publisher for the creditable manner in which the mechanical part is executed. Good type and clean white paper in a book, like clean linen, and decent clothes on a man, add much to its respectability.

